# The Deterioration of Sarcopenia Post-Transarterial Radioembolization with Holmium-166 Serves as a Predictor for Disease Progression at 3 Months in Patients with Advanced Hepatocellular Carcinoma: A Pilot Study

**DOI:** 10.3390/jpm14050511

**Published:** 2024-05-11

**Authors:** Claudio Trobiani, Nicolò Ubaldi, Leonardo Teodoli, Marcello Andrea Tipaldi, Federico Cappelli, Sara Ungania, Giulio Vallati

**Affiliations:** 1 Interventional Radiology Unit, “IRCCS Istituto Nazionale Tumori Regina Elena”, 00144 Rome, Italy; claudio.trobiani@ifo.it (C.T.); leonardo.teodoli@ifo.it (L.T.); federico.cappelli@ifo.it (F.C.); giulio.vallati@ifo.it (G.V.); 2 Department of Medical Surgical Sciences and Translational Medicine, Sapienza-University of Rome, Radiology Unit–Sant’Andrea Hospital, 1035 Via Di Grottarossa, 00189 Rome, Italy; tipaldi.andrea@gmail.com; 3 Medical Physics Department, “IRCCS Istituto Nazionale Tumori Regina Elena”, 00144 Rome, Italy; sara.ungania@ifo.it

**Keywords:** TARE, sarcopenia, HCC, interventional oncology, liver, Holmium-166

## Abstract

Purpose: The aim of this pilot study is to explore the relationship between changes in sarcopenia before and after one to three months of Transarterial Radioembolization (TARE) treatment with Holmium-166 (166Ho) and its effect on the rate of local response. Our primary objective is to assess whether the worsening of sarcopenia can function as an early indicator of a subgroup of patients at increased risk of disease progression in cases of hepatocellular carcinoma (HCC). Methods: A single-center retrospective analysis was performed on 25 patients with HCC who underwent 166Ho-TARE. Sarcopenia status was defined according to the measurement of the psoas muscle index (PMI) at baseline, one month, and three months after TARE. Radiological response according to mRECIST criteria was assessed and patients were grouped into responders and non-responders. The loco-regional response rate was evaluated for all patients before and after treatment, and was compared with sarcopenia status to identify any potential correlation. Results: A total of 20 patients were analyzed. According to the sarcopenia status at 1 month and 3 months, two groups were defined as follows: patients in which the deltaPMI was stable or increased (No-Sarcopenia group; *n* = 12) vs. patients in which the deltaPMI decreased (Sarcopenia group; *n* = 8). Three months after TARE, a significant difference in sarcopenia status was noted (*p* = 0.041) between the responders and non-responders, with the non-responder group showing a decrease in the sarcopenia values with a median deltaPMI of −0.57, compared to a median deltaPMI of 0.12 in the responder group. Therefore, deltaPMI measured three months post-TARE can be considered as a predictive biomarker for the local response rate (*p* = 0.028). Lastly, a minor deltaPMI variation (>−0.293) was found to be indicative of positive treatment outcomes (*p* = 0.0001). Conclusion: The decline in sarcopenia three months post-TARE with Holmium-166 is a reliable predictor of worse loco-regional response rate, as evaluated radiologically, in patients with HCC. Sarcopenia measurement has the potential to be a valuable assessment tool in the management of HCC patients undergoing TARE. However, further prospective and randomized studies involving larger cohorts are necessary to confirm and validate these findings.

## 1. Introduction

Primary liver cancer was diagnosed in roughly 800,000 patients worldwide in 2022, accounting for more than 700,000 deaths annually [1]. Transarterial Radioembolization (TARE) is a minimally invasive treatment that combines low-volume arterial embolization with internal radiation therapy. It is commonly used for unresectable hepatocellular carcinoma (HCC) and is generally regarded as safe and effective, showing improvements in overall survival compared to conventional treatments [2]. Initially developed as a palliative treatment in the 1960s [3], by late 2022, TARE had been incorporated into the early-stage section (i.e., BCLC 0-A) of the Barcelona Clinic Liver Cancer algorithm, particularly in cases where other HCC treatments have failed or are not feasible [4]. A recent phase II randomized trial (TRACE) comparing TARE to transarterial chemoembolization (TACE) in patients with early- or intermediate-stage HCC revealed that TARE provided superior tumor control and overall survival outcomes [5]. Compared to TACE, TARE is associated with lower toxicity to healthy liver tissue and reduced static embolization effects, potentially resulting in less hepatocyte damage and better tolerability, particularly in patients with portal vein thrombosis [6,7]. Patient selection for TARE involves evaluation by a multidisciplinary oncology team, typically considering individuals with a life expectancy of more than three months, bilirubin levels below 2 mg/dL, albumin levels above 3 g/dL, and an Eastern Cooperative Oncology Group status of 2 or lower [8].

Various radioactive isotopes are employed for Transarterial Radioembolization (TARE), with Yttrium-90 (90Y) being the most commonly utilized and the longest established option, although Holmium-166 (166Ho) microspheres have recently become available as an alternative. 166Ho microspheres received a CE mark under the commercial name of QuiremSpheres™ (Quirem BV, Deventer, The Netherlands) in 2015. The basis of 166Ho is Poly-L-lactic acid, offering distinct advantages over Yttrium-90. It possesses both high-energy beta emission for the treatment process and lower gamma emission for scoping, with the latter assessed using SPECT imaging. Additionally, due to its paramagnetic properties, MRI facilitates convenient assessment of tumor dose distribution and quantification [9,10]. Typically, 166Ho is loaded into either resin or glass microspheres, with resin being more readily available but having lower density [8]. Holmium has a relatively medium–short half-life of 26.8 h, resulting in a high dose rate over a brief period. This allows for the evaluation of treatment response through CT, FDG-PET, or MRI at shorter intervals compared to 90Y, typically <6 months in our clinical practice. This innovative treatment has seen potential benefits in improving intrahepatic distribution prediction compared with current standard treatment [9]. Up to now, Holmium microspheres have only been recently used in clinical practice; specifically, less than 10 clinical trials have been carried out [9], of which the most recent were the HEPAR Primary study, which assessed HCC [11], and the HORA EST clinical trial, which assessed early HCC [12], evaluating the efficacy and toxicity profiles.

Several prognostic staging systems have been developed for HCC; however, they often overlook patients’ performance status [13]. Sarcopenia, defined as the “progressive loss of muscle mass and strength with a risk of adverse outcomes such as disability, poor quality of life, and death” [12], manifests relatively early in HCC and can independently negatively predict HCC-related mortality in patients undergoing loco-regional treatment [13,14,15]. Indeed, sarcopenia has been associated with poor treatment response in HCC [16] and has recently been identified as a predictor of progressive disease in HCC treated with TARE-90Y [15]. Given that sarcopenia could be used to assess the overall response to loco-regional HCC treatment [15,17,18], it is crucial to identify patients with worsening sarcopenia, as they might benefit from early loco-regional re-treatment and nutritional support to restore muscle mass and strength. To the best of our knowledge, there have been no other studies evaluating a treatment response predictor, such as sarcopenia status, following TARE-166Ho for HCC.

The aim of this preliminary study is to assess whether there is a potential relationship between changes in sarcopenia before and after one–three months of TARE-166Ho treatment and the rate of local response it induces, and thus to determine if the deterioration of sarcopenia worsening can serve as an early identifier of a subgroup of patients who are at a high risk of disease progression.

## 2. Materials and Methods

### 2.1. Patients

A retrospective investigation was conducted on patients with BCLC-B large monofocal or multifocal HCC who underwent TARE-166Ho in the period 2022–2023. The sarcopenia status was defined according to the measurement of the psoas muscle index (PMI), as described later, at baseline, one month, and three months after TARE. The population was divided into two groups according to sarcopenia status: patients in which the deltaPMI was stable or increased (No-Sarcopenia group) and patients in which the deltaPMI decreased (Sarcopenia group). The radiological response, according to mRECIST criteria, was assessed one and three months subsequent to the procedure and patients were grouped into responders and non-responders. Throughout the follow-up period, patients did not modify their exercise and nutrition habits from baseline. All methods or experimental protocols were approved by the local institutional review board and were carried out in accordance with the relevant guidelines of the Declaration of Helsinki. Informed consent was collected from all participants.

### 2.2. Sarcopenia Measurement

**Figure 1:**
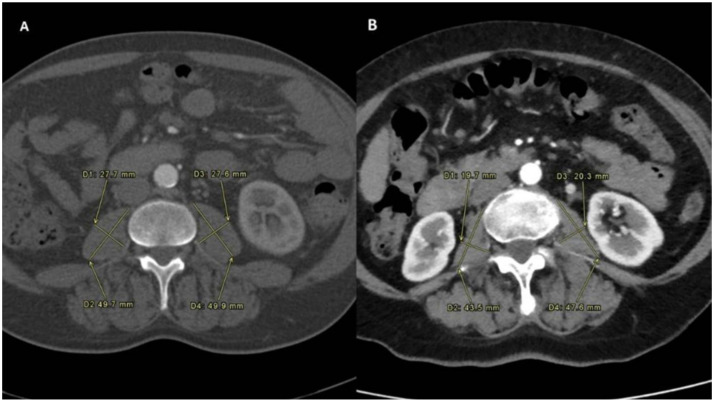
Sarcopenia status measured before TARE in the Sarcopenia group (**A**) and the No-Sarcopenia group (**B**).

**Figure 2:**
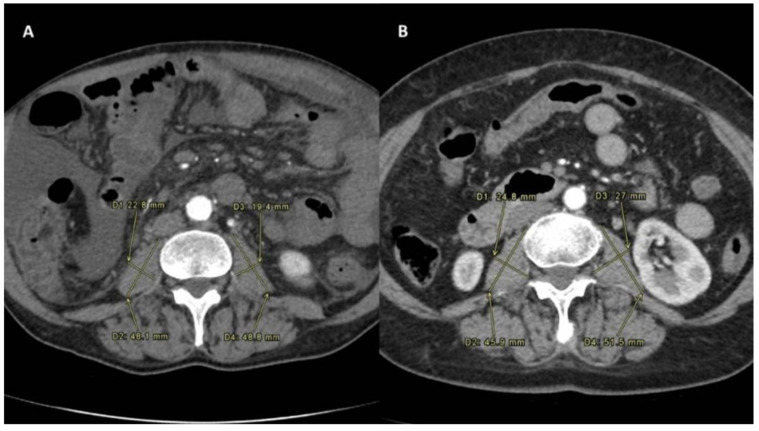
Sarcopenia status measured after TARE in the Sarcopenia group (**A**) and the No-Sarcopenia group (**B**).

Sarcopenia manifests as a syndrome marked by gradual and widespread decline in skeletal muscle mass and strength, closely associated with physical impairment, diminished quality of life, and mortality [19]. The assessment of sarcopenia status was determined by measuring the PMI before and after treatment. Sarcopenia was confirmed in patients with reduced PMI after treatment. The PMI was calculated using the following formula: PMI [mm/m^2^]: [(minor diameter of left psoas + major diameter of left psoas + minor diameter of right psoas + major diameter of right psoas)/4]/height in m^2^ [20]. All the psoas measurements were performed at the level of L3-L4 (Figure 1 and Figure 2) on multiphasic CT scans performed in the same center having all identical slice thickness.

### 2.3. TARE

All procedures were carried out by experienced interventional radiologists with more than five years of experience. For the treatment, 80–170 MBq 166mHo-loaded scout microspheres were administered slowly through a 2.7-F micro-catheter (Progreat; Terumo Europe NV, Leuven, Belgium) placed in the pathological feeder artery. The distribution of the drug was assessed using SPECT imaging. Post-therapy SPECT/CT scans (Symbia IntevoTM system; Siemens, Erlangen, Germany) were performed between 1 and 20 h after SIRT to evaluate the distribution of the microspheres thanks to 2D and 3D dosimetry maps.

Firstly, the accuracy and intensity of the 166Ho microspheres’ activity distribution were evaluated by analyzing the 2D activity intensity peak (Pixel Value) of the signal along a line crossing the treated area. A higher peak indicated a more intense signal within the targeted area.

Later, the 3D effective dose in Gy distributed to the lesion and liver parenchyma per unit cumulated activity (GBq), was calculated according to the activity distribution obtained from SPECT/CT imaging, utilizing a MIM 6.1.7 workstation (MIM Software Inc., Cleveland, OH, USA). For each patient, the mean absorbed dose (<D>) in Gy for the normal liver and tumor were compared with the expected values (<D> to tumor > 100 Gy, <D> to normal liver < 40 Gy) to assess the treatment efficacy.

### 2.4. Statistical Analysis

The Mann–Whitney U test was used for evaluating the deltaPMI according to treatment response at 1 and 3 months after TARE. The PMI at the time of TARE was compared with the PMI measurements at one and three months after TARE. The deltaPMI measurements between the first PMI measurement and the controls at one and three months were evaluated. Variables with a *p* < 0.05 were considered statistically significant.

The accuracy of the different sarcopenia measurements performed over time was assessed through c-statistics analysis, with the intent to evaluate their ability to predict progressive disease after TARE. Areas under the curve (AUCs) and 95% CI were reported. Fisher’s exact test was used for comparisons of categorical variables.

## 3. Results

Between 2022 and 2023, a total of 25 patients with BCLC-B HCC underwent TARE-166Ho. Five patients were excluded from the study due to drop-out and the absence of post-treatment follow-up CT scans. The patients’ demographics and clinical features did not differ between the two sarcopenia groups and are depicted in Table 1. Additionally, tumor characteristics at the time of TARE were similar between the two groups, in terms of maximum diameter of target nodule (*p* = 0.60), number of lesions (*p* = 0.43), and bilobar involvement of the disease (*p* = 0.84). Also, PMI at baseline was not significant between the two groups (*p* = 0.79). Lastly, the HCC biological markers, such as alpha-fetoprotein (AFP) and protein induced by vitamin K absence (PIVKA), were similar between the two groups.

Furthermore, no statistical differences were observed between the two groups in terms of liver function features or disease burden. Additionally, no disparities were found between the groups regarding administered dose activity: the mean activity administered in Gigabequerels (GBq) was 4.5 (3.60–8.82) in the Sarcopenia group and 5.1 (2.62–9.08) in the No-Sarcopenia group. The technical success of TARE was 100%, with the administration of 166Ho microspheres within the tumor-feeding artery reached in all cases. No post-procedural complications were reported in either group.

Dose activity metrics in TARE, in terms of activity intensity peak and mean absorbed dose to the tumor and to the liver, were calculated and compared between the two sarcopenia groups, as shown in Table 2. Regarding the efficacy of TARE, it demonstrated a favorable dosimetrical profile in both 2D and 3D analysis. In terms of 2D evaluation, no statistical differences were found between the groups in terms of activity intensity peak calculated in grayscale (903.8 ± 110.1 in the Sarcopenia group vs. 998.6 ± 94.9 in the No-Sarcopenia group, *p* = 0.21). For 3D dose analysis (expressed as absorbed dose in Gy), the mean absorbed dose to normal liver (<Dnliver>) was <40 Gy for both groups, with no significant differences observed (26.0 ± 5.2 Gy in the Sarcopenia group vs. 29.5 ± 9.8 Gy in the No-Sarcopenia group, *p* = 0.50).

Similarly, the mean absorbed dose to the tumor (<Dtumor>) was calculated for all patients, with no statistical differences between the groups (154.2 ± 56.7 in the Sarcopenia group vs. 162.7 ± 47.8 in the No-Sarcopenia group, *p* = 0.13). Additionally, the mean absorbed dose to normal liver (<Dnliver>) remained <40 Gy for both groups, with no significant differences (26.0 ± 5.2 Gy vs. 29.5 ± 9.8 Gy, *p* = 0.50).

According to the sarcopenia status, measured as the deltaPMI before and after treatment at 1 month and 3 months, two groups were defined as follows: patients in which the deltaPMI was stable or increased (No-Sarcopenia group; *n* = 12) vs. patients in which the deltaPMI decreased (Sarcopenia group; *n* = 8).

The radiological response rate was evaluated for all patients one month and three months after the TARE procedure, and patients were grouped as follows: those with standard and progressive disease were added to the non-responder group, and those with complete response or partial response were added to the responder group.

The local response rate was compared with the sarcopenia status at 1 and 3 months.

**Figure 3:**
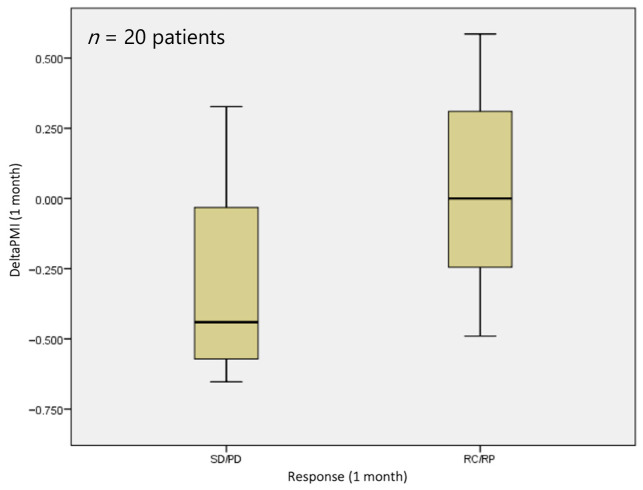
DeltaPMI after 1 month evaluated against response to treatment.

At 1 month, no statistically significant difference (*p* = 0.229) was observed in terms of sarcopenia status, with a median deltaPMI of 0 vs. 0.44 in the responder and non-responder groups, respectively (Figure 3).

**Figure 4:**
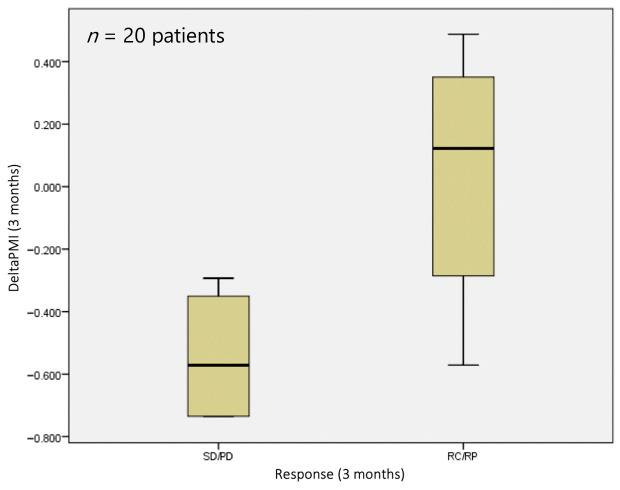
DeltaPMI after 3 months evaluated against response to treatment.

At 3 months, however, a statistically significant difference (*p* = 0.041) was observed in terms of sarcopenia status, with a median deltaPMI of 0.12 vs. 0.57 in the responder and non-responder groups, respectively (Figure 4).

A further Mann–Whitney test was conducted to investigate whether deltaPMI could be predictive of a response constant over time. For this purpose, a variable named *bestresp* was introduced and defined as follows: 1 if the response after one month was confirmed after three months and 0 otherwise.

**Figure 5:**
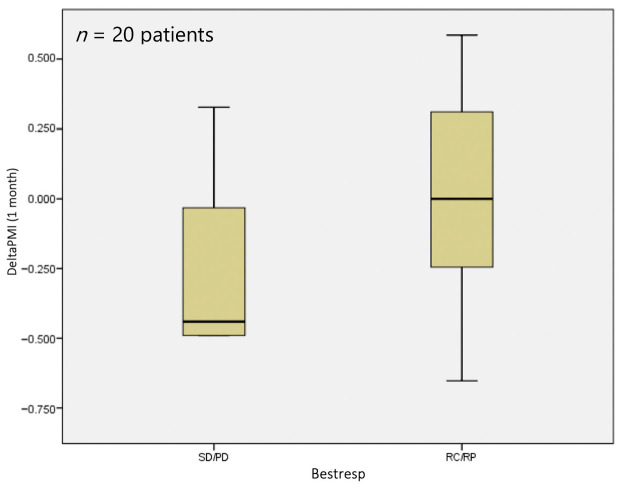
DeltaPMI after 1 month evaluated against the bestresp variable.

No statistically significant relevance (*p* = 0.247) was observed for data collected one month after TARE, probably due to the fact that it is premature to evaluate the radiological response to TARE treatment only one month after the procedure (Figure 5).

**Figure 6:**
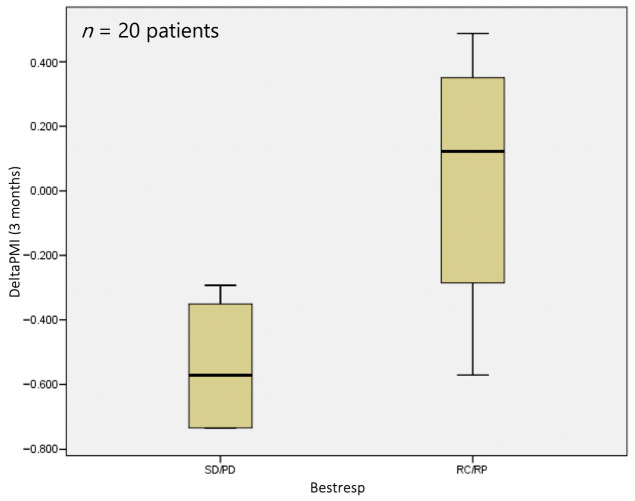
DeltaPMI after 3 months evaluated against the bestrep variable.

The same analysis conducted 3 months after TARE showed that deltaPMI can be considered to be a predictive biomarker of local response rate (*p* = 0.028). In particular, we found that a strong negative variation in PMI is associated with a poor response to treatment, with a median deltaPMI of −0.57 in the non-responder and 0.12 in the responder groups, respectively (Figure 6). It was interesting to note that the diagnostic ability for stable or progressive disease of the sarcopenia measurement increased with time after the TARE procedure.

The ROC analysis was also used to investigate the optimal cut-off of deltaPMI that is statistically relevant in the prediction of a bestresp (i.e., a response to treatment constant over time).

At three months, the deltaPMI value had an AUC = 0.875 (*p* = 0.0001, CIs 0.617 to 0.984). In particular, a PMI variation greater than −0.293 was associated with a bestresp.

This seems to confirm that a small deltaPMI variation is predictive of good treatment. In fact, all 10 patients (100%) who showed a PMI variation greater than the cut-off had a good response to treatment (Fisher’s exact test, *p* = 0.019).

## 4. Discussion

Sarcopenia deterioration at three months after TARE treatment with Holmium-166 is a reliable predictor of worse loco-regional response rate in HCC patients.

Sarcopenia has functioned as a predictive element across various clinical scenarios. It has been employed as a predictive factor among patients with HCC [21], including patients undergoing Sorafenib [22,23] or intra-arterial chemoembolization [20], and those pre- or post-transplant [24]. Recently, sarcopenia worsening after TARE treatment with Yttrium-90 has been associated with progressive disease in the early stages [15]. Lastly, with this study, we have determined the association of sarcopenia deterioration at 3 months with TARE-166Ho.

The latest development in radionuclide therapy, Holmium-166, has emerged as a viable substitute for Yttrium-90 in TARE, demonstrating several advantages as previously described. The analysis conducted three months after TARE-166Ho treatment revealed that sarcopenia status could serve as a predictive biomarker for the local response rate (*p* = 0.028). Importantly, our results showed that a decrease in PMI was associated with an unfavorable treatment response, with a median deltaPMI of −0.57 observed in the non-responder group compared to a median value of 0.12 in the responder cohort (*p* = 0.041). Furthermore, we noted an increasing trend in the diagnostic efficacy of sarcopenia measurement for distinguishing stable or progressive disease over the one-to-three-month period following TARE.

This highlights the significance of sarcopenia assessment in effectively predicting the intermediate local outcome following TARE-166Ho, irrespectively of gender differences. Consequently, the deterioration of sarcopenia correlates with a poorer mRECIST score at the intermediate stage.

No statistically significant differences in sarcopenia status were observed at one month post-procedure in either group. This lack of difference is likely due to the premature nature of assessing radiological response to TARE treatment within just one month after the procedure. Additionally, there were no statistically significant distinctions in pre-procedural sarcopenia status between the cohort of patients experiencing progressive disease and those achieving complete response, partial response, or stable disease. This suggests that the initial evaluation of sarcopenia cannot reliably predict unfavorable oncological outcomes following TARE.

Of note, our assessment of TARE treatment response was carried out through CT and MRI or FDG-PET scans, techniques which necessitate a minimum of three–six months to allow for accurate insights into TARE outcomes [25].

In addition to what was previously published regarding the association between sarcopenia worsening and HCC progression at 1 and 3 months after TARE-90Y, this study confirms the same outcomes utilizing Holmium-166 [13]. However, Holmium releases a higher dose rate over a briefer period compared to Y-90, obtaining a faster tumor response and potentially affecting the muscle volume sooner. This allows sarcopenia to be used as a faster predictive index for tumor response.

The divergence in sarcopenia status, identified three months post-procedure through routinely conducted multiphasic CT scans, emerges as a convenient non-invasive predictive biomarker for treatment efficacy, confirming the association between skeletal muscle depletion and a poorer loco-regional response rate. These findings suggest that patients could benefit from early adjustment for skeletal muscle depletion using simple non-invasive methods, which could enhance clinical management by reducing the likelihood of disease progression. Early identification of declining sarcopenia could prompt the implementation of aggressive support protocols. Multimodal approaches, including nutritional support, physical therapy, and medications such as ibuprofen (Menac trial) and selective androgen receptor modulators, as well as ghrelin receptor activation with agonists, are recognized as foundational in managing sarcopenia.

Moreover, the initial sarcopenic status could serve as a guiding factor in selecting a subset of HCC patients undergoing TARE as a preparatory step for liver transplant. This suggests that sarcopenia status could assist in identifying individuals with predictable positive outcomes following transplantation who may benefit from it. Conversely, the early identification of patients at risk of non-responsiveness could be valuable in guiding the timely implementation of systemic therapies. Moreover, the potential of sarcopenia status to predict disease progression could assist in identifying candidates for early TARE re-treatment. Discussions regarding re-treatment often arise in cases where patients tolerated the initial procedure without substantial improvement, though there is no consensus regarding the optimal timing for re-treatment, especially considering the necessary CT/MRI assessment time-frame.

The study has several limitations that need discussion. Foremost, the retrospective nature of the study design precludes the establishment of a randomized framework, which may influence the generalizability of the findings. Secondly, the sample size under analysis was relatively modest; however, given the encouraging results of this pilot study, we hope to continue with a prospective study at our institution, focusing on 166Ho microspheres and sarcopenia, involving a larger sample size. The future trajectory of research would ideally also encompass randomized studies to confirm and further validate these results.

## 5. Conclusions

TARE treatment has firmly established itself as a therapeutic option for patients with locally advanced HCC; more recently, Holmium-166 has introduced new possibilities in this population due to its various advantages. However, assessing the response to TARE treatment in the early stages of follow-up can be challenging. The deterioration of sarcopenia status is a reliable predictor of a worse local response rate at three months after TARE-166Ho, potentially serving as a valuable indicator for identifying patients at early high risk of disease progression. Indeed, the PMI decreased significantly more in the group of poor responders. If our findings are confirmed in larger populations, they could play a pivotal role in the early identification of patients who do not respond to endovascular treatment with Holmium-166 and who may benefit from prompt integrative restorative interventions.

## Figures and Tables

**Table 1:** Patient population demographics and clinical characteristics.

Variable	No-Sarcopenia Group (*n* = 12)	Sarcopenia Group (*n* = 8)	*p*
	Median (IQR) or *n* (%)		
Age (years)	63 (58–67)	63 (57–70)	0.44
Male sex	7 (58.3)	5 (62.5)	0.78
Height (cm)	170 (162–180)	174 (165–180)	0.32
Ascites (any grade) Moderate	4 (33.3) 1 (8.3)	2 (25) 1 (12.5)	0.78
Maximum size of treated lesion (mm)	45 (33–71)	50 (35–78)	0.60
Number of lesions	2 (1–4)	3 (2–4)	0.43
Bilobar involvement	7 (58.3)	4 (50)	0.84
Liver parenchyma involved > 50%	3 (25)	2 (25)	1.00
Bilobar TARE treatment	7 (58.3)	4 (50)	0.67
AFP measure (ng/mL) pre-procedural	58 (19–235)	69 (26–210)	0.85
PIVKA measure (AU/mL) pre-procedural PMI (mm/m^2^) at baseline	155 (93–436) 12 (6–16)	172 (44–445) 12 (8–13)	0.89 0.79

Abbreviations: *n*, number; IQR, interquartile ranges.

**Table 2:** Comparison of dose activity metrics in TARE between Sarcopenia and No-Sarcopenia groups.

	Sarcopenia Group n = 8	No-Sarcopenia Group n = 12	*p*
Activity intensity peak (Grayscale)	903.8 ± 110.1	998.6 ± 94.9	0.21
<Dtumor> (Gy)	154.2 ± 56.7	162.7 ± 47.8	0.13
<Dnliver> (Gy)	26.0 ± 5.2	29.5 ± 9.8	0.40

## Data Availability

The datasets generated during and/or analyzed during the current study are available from the corresponding author on reasonable request.
